# The decay of motor adaptation to novel movement dynamics reveals an asymmetry in the stability of motion state-dependent learning

**DOI:** 10.1371/journal.pcbi.1005492

**Published:** 2017-05-08

**Authors:** Eghbal A. Hosseini, Katrina P. Nguyen, Wilsaan M. Joiner

**Affiliations:** 1 Department of Bioengineering, Sensorimotor Integration Laboratory, George Mason University, Fairfax, VA United States; 2 Department of Brain and Cognitive Sciences, Massachusetts Institute of Technology, Cambridge, MA United States; 3 Department of Biomedical Engineering Carnegie Mellon University, Pittsburgh, PA United States; 4 Krasnow Institute for Advanced Study George Mason University, Fairfax, VA United States; Johns Hopkins University, UNITED STATES

## Abstract

Motor adaptation paradigms provide a quantitative method to study short-term modification of motor commands. Despite the growing understanding of the role motion states (e.g., velocity) play in this form of motor learning, there is little information on the relative stability of memories based on these movement characteristics, especially in comparison to the initial adaptation. Here, we trained subjects to make reaching movements perturbed by force patterns dependent upon either limb position or velocity. Following training, subjects were exposed to a series of error-clamp trials to measure the temporal characteristics of the feedforward motor output during the decay of learning. The compensatory force patterns were largely based on the perturbation kinematic (e.g., velocity), but also showed a small contribution from the other motion kinematic (e.g., position). However, the velocity contribution in response to the position-based perturbation decayed at a slower rate than the position contribution to velocity-based training, suggesting a difference in stability. Next, we modified a previous model of motor adaptation to reflect this difference and simulated the behavior for different learning goals. We were interested in the stability of learning when the perturbations were based on different combinations of limb position or velocity that subsequently resulted in biased amounts of motion-based learning. We trained additional subjects on these combined motion-state perturbations and confirmed the predictions of the model. Specifically, we show that (1) there is a significant separation between the observed gain-space trajectories for the learning and decay of adaptation and (2) for combined motion-state perturbations, the gain associated to changes in limb position decayed at a faster rate than the velocity-dependent gain, even when the position-dependent gain at the end of training was significantly greater. Collectively, these results suggest that the state-dependent adaptation associated with movement velocity is relatively more stable than that based on position.

## Author summary

Human motor adaptation of limb movement in response to force perturbations has been shown to be motion-state dependent. That is, the compensatory response to these disturbances is correlated and proportional to the temporal changes in the position, velocity, and acceleration during the motion. Despite a growing understanding of this adaptation process, there is little information on the relative stability of this learning when based on these different temporal features of movement. Here we modified a previous computational model of motor adaptation to predict the decay of the compensatory response associated to different motion states, specifically learning based on temporal variations in limb position and velocity. We confirmed the simulated behavior by examining the decay of the temporal force output after subjects were trained to compensate for movement disturbances based on different combinations and magnitudes of these two motion states. Both simulation and behavioral results show that velocity-based learning decays at a slower rate than position-based, even when learning is significantly biased towards the latter at the end of training. Collectively, these results suggest that motion-state learning based on movement velocity is more stable than that based on limb position.

## Introduction

The motor system adapts to movement perturbations, a process largely driven by the error between the executed movement and the predicted consequences of that movement [1–3]. This short-term form of motor learning is a gradual updating of the motor commands required to counteract the movement perturbation. Similar to learning, the decay of adaptation following the removal of the perturbation is typically a gradual process as the motor commands revert back to the state prior to exposure [4,5]. Thus, examining and comparing the progression and decay of motor adaptation provides insight into the stability of these updates to the issued motor commands.

The decay of motor adaptation has been studied for various behavioral paradigms involving limb movement: prism displacement [6,7], locomotion [8,9], visuomotor alterations [5,10,11] and force-field perturbations [12–15] In the last case, subjects make reaching movements while interacting with a robotic manipulandum and are exposed to a force perturbation typically dependent upon either a single motion kinematic parameter (e.g., changes in position, velocity or acceleration during the movement) or the combination of these motion states [16]. In response to the movement disturbance, subjects apply an adaptive response based on the temporal characteristics of the limb state. Although previous investigations of force-field adaptation have examined the time course and the factors that influence the stability and retention of these state-dependent compensatory responses [4,12,14,17–23] the relative stability of the different state-dependent components that drive adaptation is not well understood, especially in direct comparison to the initial learning process.

Here, we applied a framework developed by Sing and colleagues [16] to compare the progression and decay of state-dependent adaptation in response to different types of novel movement dynamics. Based on this framework, the feedforward motor output in response to the applied force perturbation is the weighted sum of gains assigned to the kinematic parameters of the reaching motion (changes in limb position and velocity, [16,23–25]). The model predicted that the changes in these gains during adaptation would follow a different time course than during the adaptation decay, but the authors did not explicitly test this prediction nor the relative stability of the changes based on the kinematic parameters. To assess the difference in the relative stability of the motor memory based on changes in limb position or velocity we first modified the original model proposed by Sing et al. [16] based on observed differences in the retention of adaptation in response to purely velocity- or position-dependent disturbances. The resulting model simulations predicted that when motor learning is based on the combination of position and velocity the decay of adaptation is biased towards velocity, independent of the final adaptation level. That is, the model predicted that the decay of position-based adaptation would occur at a faster rate, even when this learning was significantly higher at the end of training. We tested two additional groups of subjects and found that the behavioral results were in agreement with the predictions of the model. Collectively, our behavioral and simulation results suggest that (1) the decay of motor adaptation is not merely the reversal of the learning process, but at least a partially distinct process likely involving separate mechanisms, (2) the velocity-based contribution to updating motor commands is more stable than that based on position, and (3) a model with asymmetrical retention factors for position- and velocity-based motor primitives can predict the time course of adaptation decay for various state-dependent motor learning goals.

## Results

### Stability of adaptive responses to position- and velocity-dependent force-field training

**Fig 1:**
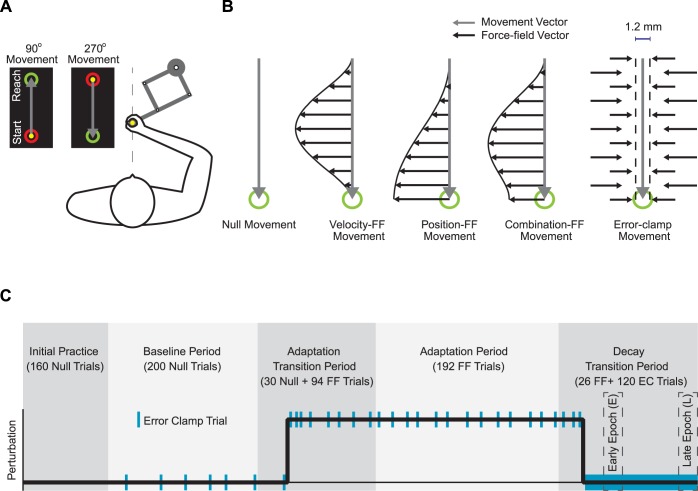
**(A) Experimental setup.** Subjects made reaching movements from mid-line in both forward (90°) and backward (270°) directions, using a robotic manipulandum. The location of the hand was represented by a filled yellow circle, while the view of arm was occluded. **(B) Trial Types.** Null movements (grey arrows) were made in the absence of the any force from the robot. During force-field trial movements, the robot applied forces that were dependent on a single or combination of motion kinematics. During velocity-dependent force-field movements the manipulandum applied lateral forces that scaled with movement velocity (black arrows). For position-dependent movements, the lateral force scaled with hand position with respect to the start position. Lastly, for both unbiased and position biased combination force-field movements the lateral force scaled with both hand position and velocity. During error-clamp movements the manipulandum constrained the movement trajectory between the two targets by countering any lateral motions. **(C) Experimental Paradigm.** Subjects first completed a baseline period, during which they experienced null movements with sparse instances of error-clamp movements (blue bars). The 1^st^ transition period, contained an initial period of null movements, followed by the abrupt application of the force-field. The adaptation period contained only force-field and error-clamp trials. Finally, the 2^nd^ transition period started with force-field movements followed by only error-clamp trials (thick blue bar). The frequency of error clamp trials increased during the two transition periods.

**Fig 2:**
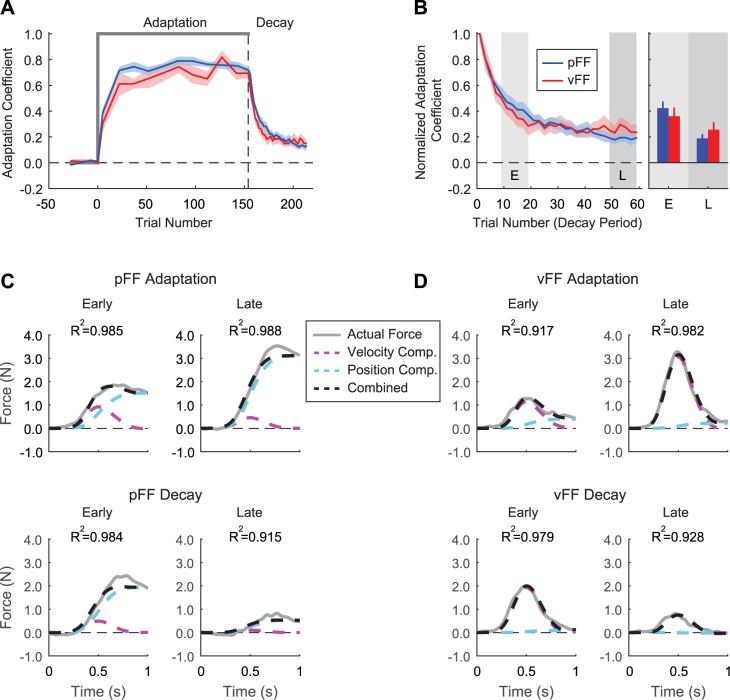
**Adaptation and subsequent decay for training in position- and velocity -dependent force-fields (A).** Comparison of the adaptation coefficients during position (pFF, blue curve) and velocity-dependent force-field training (vFF, red curve). Each point during the adaptation period is the average adaptation coefficient across subjects for windows of 10 or 15 trials. During the decay period, the points are average across all subjects for each trial. The start of the decay period is shown as a vertical dashed line. Shaded areas show standard error. **(B)** Normalized decay for pFF and vFF training. The adaptation coefficients were scaled with respect to the first coefficient in the decay period, with the first point rescaled to a value of 1.0. Bar graphs show the average adaptation across subjects for early and late epochs of the decay, represented by the shaded areas. Error bars are standard error. **(C and D)** Temporal force profiles during adaptation and decay for pFF and vFF training. Top panel shows the evolution of the force patterns in the early and late stages of adaptation, while the bottom panel shows the changes in early and late stages of the decay period. The average force across all subjects is shown by a gray trace. The contribution of position and velocity to the force profile is represented by the blue and pink dashed lines. The combination of the position and velocity contribution is shown by a black dashed line, and approximates actual exerted forces. The R^2^ value from the regression between actual (thick gray trace) and combined position-velocity fit (black dashed trace) is provided in the top left of each panel.

We first trained subjects to make reaching movements in either a position- or velocity-dependent force-field (pFF and vFF) (Fig 1). Each subject experienced only one type of perturbation after an initial baseline period, during which error-clamp trials were used to quantify the feedforward adaptive changes to the motor output (see Materials and Methods). Based on the forces subjects applied during the error-clamp trials, we were able to determine the adaptation coefficient (the linear regression of the applied lateral force profile onto the ideal compensatory force profile) and the respective gain of the position-dependent and velocity-dependent force components to the overall force profile (see Materials and Methods). Fig 2A plots the adaptation coefficient as a function of trial number for pFF and vFF training. Similar to previous studies [16,21,26,27], we observed a fast progression of adaptation early on (within the first 15 trials) that plateaued after approximately 75 trials for both force-field types (Fig 2A). An exponential fit of the adaptation curve showed a faster overall adaptation for pFF training (time constant of 7.2 ± 0.8 for pFF compared to 12.3 ± 5.5 trials for vFF. See S1A Fig). The amount of adaptation at the end of training was significantly greater than at the beginning, but the adaptation levels were not significantly different between perturbation types (2-way ANOVA, *P* < 0.001 for the main effect of training period and *P* = 0.22 for the main effect of perturbation type). Specifically, early adaptation levels were not significantly different between pFF and vFF training (0.38 ± 0.03 compared to 0.30 ± 0.05, mean ± SEM, *P* = 0.18, two-tailed t-test). There was also no difference in the adaptation level between vFF and pFF late in training where the behavior asymptotes (0.72 ± 0.02 for pFF and 0.69 ± 0.04 for vFF, *P* = 0.58, two-tailed t-test). (Early adaptation period was determined over trials 1–15, while the late/asymptotic adaptation period was trials 150–160. See Materials and Methods for justification of these ranges.)

Immediately following training, subjects experienced a sequence of consecutive error-clamp trials to determine the decay of feedforward changes to motor output. Following the start of the consecutive error clamps the adaptation coefficient began to decay and reached asymptote by the end of the period. An exponential fit of the adaptation decay curve showed a faster decrease in adaptation for vFF over pFF training (time constant of 11.0 ± 2.2 trials for pFF compared to 7.1 ± 1.0 trials for vFF. See S1B Fig). The adaptation coefficient levels at the end of the decay period remained significantly greater than baseline levels (vFF: 0.006 ± 0.006 vs. 0.26 ± 0.05, pFF:-0.004 ± 0.004 vs. 0.19 ± 0.03, paired two tailed t-test, *P* < 0.001 for both cases). Although the pattern of decay was similar for both pFF and vFF training (Fig 2A), there was a slight, but insignificant difference in the adaptation level before the start of decay as noted above. In order to examine the decay with respect to the final adaptation levels, we normalized the decay of adaptation by the initial value of the adaptation coefficient at the beginning of the decay period (Fig 2B). Starting at an adaptation level of 1.0, we analyzed the decay of adaptation in early and late epochs during the decay period. The percentage of adaptation at the beginning of the decay period was significantly greater than at the end, but the adaptation levels were not significantly different between perturbation types (2-way ANOVA, *P* < 0.001 for the main effect of decay period and *P* = 0.97 for the main effect of perturbation type). In the early epoch, there was not a significant difference in the percentage of adaptation that remained for pFF and vFF training (pFF: 42.3 ± 5.2%, vs vFF: 36.0 ± 6.2%; *P* = 0.41, post hoc comparisons using Bonferroni correction). This was also true for the late epoch (pFF: 18.8 ± 3.4%, vs vFF: 25.5 ± 5.3%; *P* = 0.35, Bonferroni correction). (The early epoch of decay was over trials 11–20, while the late epoch was trials 50–60. See Materials and Methods for justification of these ranges.)

Although the one dimensional adaptation coefficient suggested similar behavior for vFF and pFF training, we were interested in the temporal characteristics of the corresponding force profiles during the adaptation and the decay periods (see Materials and Methods). As in previous studies [16,23,25] we compared the temporal shape of the force profiles with changes in limb position and velocity (Fig 2C and 2D). As shown previously [16], early in adaptation the force pattern was dependent on both the position and velocity changes during the movement (pFF: 21.5 ± 2.2% for velocity and 78.5 ± 2.2% for position, vFF: 67.8 ± 7.3% for velocity and 32.2 ± 7.3% for position) (top panels in Fig 2C and 2D). Notably, late in the adaptation period the force pattern was mostly aligned with the appropriate movement parameter for the adaptation. In other words, the force exerted by subjects in the late phase of pFF adaptation was largely aligned with changes in limb position (95.9 ± 0.6% compared to 4.1 ± 0.6% for velocity), and late adaptation to vFF was mostly aligned with changes in movement velocity (93.2 ± 1.6% compared to 6.8 ± 1.6% for position).

We also examined the temporal force patterns during the decay period of the respective force-field perturbations. Interestingly, the force profiles remained aligned to the appropriate motion state required to compensate for the perturbation in both the early and late stages of decay (bottom panels in Fig 2C and 2D). In the early phase of the decay of pFF learning (Fig 2C bottom panel), the force profiles mainly consisted of a position-dependent component with a minimal velocity-dependent component (90.1 ± 7.0% compared to 9.9 ± 7.0%). In the late decay phase of pFF learning, the position-dependent component continued to contribute the most to the exerted force while the velocity contribution remained small (80.4 ± 8.0% compared to 19.6 ± 8.0%). Similarly, the force profiles in both the early and late decay phases of vFF learning were mostly dependent on movement velocity, with less contribution of limb position (early: 76.4 ± 7.0% compared to 23.6 ± 7.0%, late: 76.0 ± 8.2% compared to 24.0 ± 8.2%) (bottom panels in Fig 2D). Thus, the comparison of the temporal force profiles suggests that the proportional contributions of limb position and velocity to the overall motor output achieved at the end of training were largely maintained during the decay of the motor learning.

### Gain-space analysis of adaptive responses to single state-dependent force-field training

**Fig 3:**
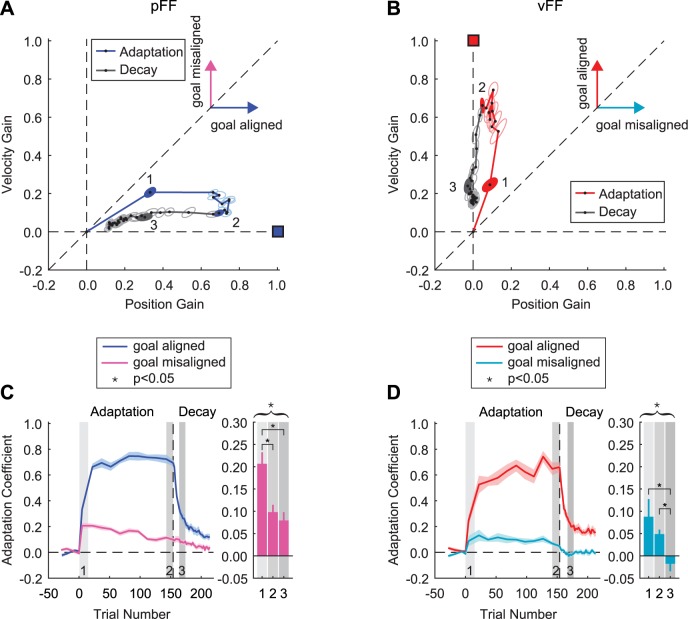
Gain-space representation of adaptation and decay for training in position- and velocity-dependent force-fields. **(A)** Evolution of position- and velocity-dependent gains during adaptation and decay for pFF training. Gain-space trajectories during adaptation and decay periods are shown in blue and gray, respectively, and are averaged across all subjects. The adaptation goal is shown as a blue filled square. Each point in the trajectory has a contribution that is goal-aligned and goal-misaligned, as shown by the two vectors. Three points were selected to compare the gains of the goal-aligned and misaligned components, labeled by the filled ellipses and the numbers 1, 2, and 3. Points 1 and 2 were early and late in the adaptation period, and were the same for both force-field types (training trials 1–15 and 150–160). Point 3 represents the average over a two trial window during the decay period at which the adaptation coefficient for the goal-aligned component was not significantly different from the early learning value (trials 12–14 and 16–18 of the decay period for pFF and vFF, respectively). Ellipses show standard error. **(B)** Evolution of position- and velocity-dependent gains during adaptation and decay for vFF training. Gain-space trajectories during adaptation and decay are shown in red and gray, respectively. The goal of adaptation is represented as a red filled square. Direction of the goal-aligned and goal-misaligned components are shown as red and cyan vectors, respectively. **(C and D)** The evolution of the goal-aligned and goal-misaligned components during training and decay are shown for each force-field perturbation. Each point during the adaptation period is the average across subjects for windows of 10 or 15 trials. Shaded regions show the standard error. Bar graphs show the amplitude of the goal-misaligned component during the periods that are highlighted in panels A and B as 1, 2, and 3. The asterisk above the bar graph represents the result of the ANOVA across the goal-misaligned components at points 1, 2, and 3. Error bars show standard error of gains for each point.

Differences in the force profile described above suggest that the gain associated to the respective motion states is different not only between the two types of force-field adaptations, but also between the learning and decay periods. In order to visualize these differences, we examined the changes in the respective gain associated to the motion states for adaptation and decay in a two dimensional gain-space (see Materials and Methods). We parsed the position-dependent and velocity-dependent force components and found a clear separation between adaptation and decay paths for both pFF and vFF training (Fig 3A and 3B). For both types of perturbations, we identified a goal-aligned and a goal-misaligned component. The goal-aligned component for pFF training is parallel to abscissa in gain space and represents the position-dependent force component, whereas the goal-misaligned component is parallel to the ordinate and represents the velocity-dependent force component. These relationships are reversed for vFF training with the goal-aligned and goal-misaligned components represented by the velocity- and position-dependent axes, respectively. In both pFF and vFF training, the goal-aligned force component had a significantly greater contribution to the initial adaptation than the goal-misaligned components (pFF: goal-aligned (0.33 ± 0.03) vs goal-misaligned (0.21 ± 0.02); vFF: goal-aligned (0.25 ± 0.03) vs goal-misaligned (0.09 ± 0.04), *P* < 0.05 in both cases). (Note there is a larger goal-misaligned component for initial pFF adaptation than for initial vFF adaptation. That is, the velocity-dependent contribution to the adaptive response for pFF training was larger than the position-dependent contribution for vFF training. This asymmetry supports an initial adaptation bias towards velocity-dependent learning as discussed below). As subjects continued to experience the force-field, contributions from the goal-aligned component increased whereas the goal-misaligned component decreased (Fig 3A and 3B). We examined two time points during training (points 1 and 2) which represent early (training trials 1–15) and late adaptation (training trials 150–160) respectively. By the end of the training period (the point labeled 2 in Fig 3A and 3B), the majority of the compensatory force was due to the contribution of the goal-aligned force component (vFF: goal-aligned (0.66 ± 0.04) vs goal-misaligned (0.05 ± 0.008); pFF: goal-aligned (0.69 ± 0.02) vs goal-misaligned (0.1 ± 0.01), *P* < 0.05 in both cases). This decrease in the goal-misaligned component resulted in a curvature in the learning trajectories (note the difference between points labeled 1 and 2). However, the magnitude of this curvature was not the same for pFF and vFF training; the contribution of the velocity-dependent force for pFF training from early (point 1 in Fig 3A) to late adaptation (point 2 in Fig 3A) was significantly different in magnitude (1^st^ point: 0.21 ± 0.02 vs. 2^nd^ point: 0.10 ± 0.01, *P* < 0.05). Although there was a similar decrease in the contribution of the position-dependent forces from early to late adaptation for vFF training, this decrease in magnitude was not significant (1^st^ point: 0.09 ± 0.04 vs. 2^nd^ point: 0.05 ± 0.01, *P* = 0.32).

The gain-space trajectories diverge from the initial adaptation path with the start of the decay period (Fig 3A and 3B gray lines). In both cases, the direction of change in gain is toward the origin of the gain-space. However, the gain-space trajectories never return completely to the origin, indicating only partial decay of the force-field adaptation within the period examined. This is in agreement to the asymptotic behavior seen at the end of the decay period for the adaptation coefficient (Fig 2A and 2B). Separation of the adaptation and decay gain-space trajectories for both pFF and vFF training demonstrate a difference in the behavior of the motor system during the decay of adaptation. The change in the goal-misaligned component between adaptation and decay dictates the shape of this separation. Fig 3C shows the gains for both the aligned and misaligned components for pFF training during the adaptation and decay period as a function of trial. The gain applied to the aligned component at the end of the decay period remained significantly greater than baseline levels, but this was not the case for the misaligned gain (aligned: -0.006 ± 0.004 vs. 0.12 ± 0.02, paired two tailed t-test, *P* < 0.001; misaligned: 0.009 ± 0.005 vs. 0.03 ± 0.02, paired two tailed t-test, *P* = 0.27). In order to capture the changes in the goal-misaligned component, we defined a third point in the gain-space trajectory. This 3^rd^ point was the trial range during the decay period at which the gain of the aligned component was not significantly different from the respective gain during initial learning (trials 12–14 and 16–18 of the decay period for pFF and vFF, respectively). For example, for pFF training, there was no significant difference in the gain for the goal-aligned component between the 1^st^ and 3^rd^ points (0.33 ± 0.03 compared to 0.27 ± 0.03, *P* = 0.16, two-tailed t-test). We determined this point in order to isolate changes in the gain of the goal-misaligned component between adaptation and decay, and quantify the trajectory separation. For pFF training the goal-misaligned component was significantly different between the three different points (ANOVA, *P* < 0.001 for the main effect of period). The value of the goal-misaligned component at the 1^st^ point was significantly greater than the respective gain at the 2^nd^ and 3^rd^ points (0.21 ± 0.02 compared to 0.09 ± 0.01 and 0.08 ± 0.02, *P* < 0.05 for both cases, multiple comparisons corrected) (Fig 3C). The difference between the 1^st^ and 2^nd^ points shows that the early adaptation level is less specific to the goal in comparison to late adaptation. The difference between the 1^st^ and 3^rd^ points further shows that for similar values of the goal-aligned component, adaptation and decay gain-space trajectories are significantly distinct. The goal-misaligned component was significantly different from zero for all 3 points, indicating that both adaptation and decay are confined in the 1^st^ quadrant of the gain-space (*P* < 0.05, two-tailed t-test).

The behavior of the goal-misaligned component was slightly different for vFF training but the overall effect was the same. As above for pFF training, we compared the adaptation and decay gain-space trajectories at points where there was no significant difference in the gain of the goal-aligned component (between the 1^st^ and 3^rd^ points in Fig 3B, 0.24 ± 0.03 compared to 0.21 ± 0.03, *P* = 0.16, two-tailed t-test). Again, the goal-misaligned component was significantly different between the three different points (ANOVA, *P* < 0.05 for the main effect of period). Unlike pFF training, there was no significant difference in the gain of the goal-misaligned component between the 1^st^ and 2^nd^ points (0.09 ± 0.04 compared to 0.05 ± 0.01, *P* = 0.52, multiple comparisons corrected). Additionally, the value of goal-misaligned component at the 3^rd^ point was significantly less than the respective gain at the 1^st^ and 2^nd^ points (-0.02 ± 0.01 compared to 0.09 ± 0.04 and 0.05 ± 0.01, *P* < 0.05 for both cases, multiple comparisons corrected) (Fig 3D). The goal-misaligned component was significantly different from zero for both early and late adaptation indicating that adaptation was confined to the 1^st^ quadrant of the gain-space, but late decay showed a nominal, but negative gain for the position-dependent component. Similar to pFF training, the gain for the goal-aligned component at the end of the decay period remained significantly greater than baseline levels (0.008 ± 0.005 vs. 0.16 ± 0.03, paired two tailed t-test, *P* < 0.001 for both cases), but the misaligned component was not (-0.003 ± 0.003 vs. 0.002 ± 0.01, paired two tailed t-test, *P* = 0.75).

Although a separation between adaptation and decay was present in both vFF and pFF training gain-space trajectories, the shapes of the trajectories were not the same. We identified three differences between the gain-space trajectories. First, the initial learning for pFF training was less specific compared to vFF adaptation. That is, vFF adaptation was more aligned with the goal (parallel to the ordinate) compared to pFF training (parallel to the abscissa). Another way to quantify this difference is to determine the angle between the learning gain-space trajectory and the ideal (straight) trajectory to the adaptation goal. For early training (1^st^ point) this angle was significantly greater for pFF training compared to vFF adaptation (pFF: 31.9° ± 2.6° vs. vFF: 18.6° ± 6.9°, *P* < 0.05, one-tail t-test). In other words, initial vFF training was more aligned with the learning goal (parallel to the velocity-dependent axis) than initial pFF adaptation (parallel to the position-dependent axis). Second, there was greater change in the learning gain-space trajectory for pFF training—a significantly larger difference was observed along the goal-misaligned gain axis between early and late adaptation for pFF adaptation (a difference in gain of 0.1 ± 0.02 for pFF compared to a difference of 0.03 ± 0.04 for vFF, two-tailed t-test, *P* < 0.05). Finally, although the decay of the goal-aligned component was slightly faster for vFF training (time constant of 10.4 ± 2.2 trials for pFF compared to 7.9 ± 0.9 trials for vFF. See S2A Fig), there was a much larger difference in the decay of the goal-misaligned component, with levels for pFF training significantly greater than vFF adaptation throughout the decay period (S2B Fig). In other words, velocity-based learning persisted at a nonzero value during the decay of pFF training. However, any subsequent position-based learning quickly decreased to zero for vFF training.

### Symmetric and asymmetric viscoelastic primitive model for motor adaptation

We hypothesized that these asymmetries for pFF and vFF training represent a possible intrinsic bias of the motor system to (1) associate the imposing perturbation with the kinematics of the movement and (2) retain the motion based learning. If the association between movement kinematics and the force-field perturbation is biased toward the velocity changes during the movement then adaptation to a force-field perturbation that is equally dependent on both position and velocity should be biased toward the velocity-dependent axis. This effect should also persist during the decay of the adaptation if velocity-dependent learning is more stable than that based on position. Sing et al. [16] previously studied adaptation to different force-field perturbations that were dependent on a combination of changes in limb position and velocity. However, the decay of this adaptation to different learning goals was not examined. Moreover, in their viscoelastic primitive model to describe the adaptation there was the basic assumption that motor learning based on changes in movement position and velocity is symmetric—an assumption challenged by the results described above. We therefore modified this model in order to make predictions about adaptation behavior and decay to novel movement dynamics dependent on different combinations of changes in limb position and velocity.

**Fig 4:**
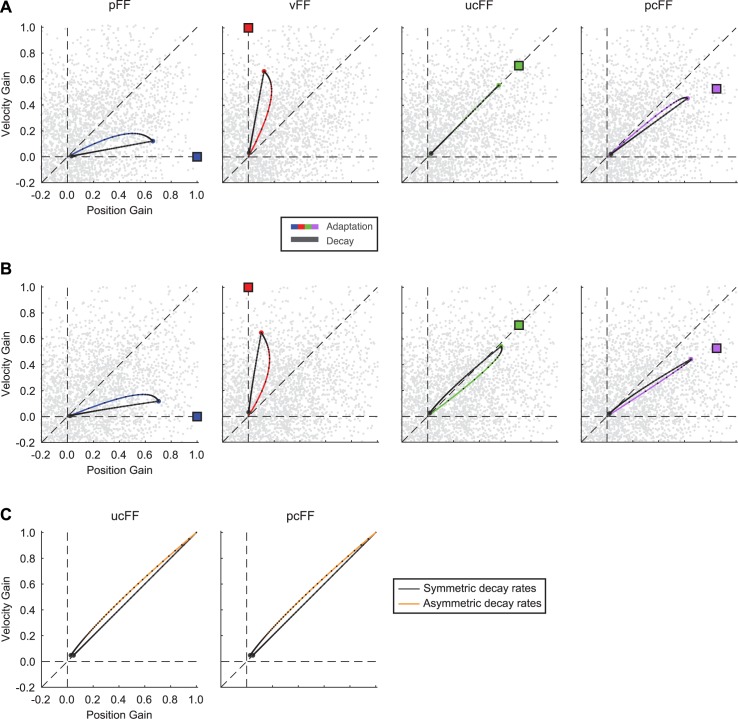
Simulation of symmetric and asymmetric viscoelastic primitive models for adaptation to different force-field types. Simulation of the gain-space trajectories for the **(A)** symmetric and **(B)** asymmetric viscoelastic primitive models fit simultaneously to the vFF and pFF behavioral data (Symmetric model: *α*_*K*_ = *α*_*B*_ = 0.951, *σ*_*K*_ *= σ*_*B*_ = 0.401, *η* = 1.5 x 10^−4^, *ρ* = 0.51. Asymmetric model: *α*_*K*_ = 0.942, *α*_*B*_ = 0.951, *σ*_*K*_ = 0.464, *σ*_*B*_ = 0.379, *η* = 1.5 x 10^−4^, *ρ* = 0.47). Adaptation and decay to pFF and vFF, and predictions of behavior for ucFF, and pcFF force-field training are depicted by the colored and gray traces, respectively. **(C)** Normalized decay of position- and velocity-dependent gains for the predictions of the symmetric (black trace) and asymmetric (orange trace) primitive models for ucFF and pcFF training.

The viscoelastic primitive model proposed by Sing et al. [16] captured the changes in the temporal pattern of force during adaptation to different types of force-field perturbations. The application of this model to the vFF and pFF behavioral data are shown in Fig 4A. In this model the force pattern in each trial is a weighted sum of motor primitives that are differentially tuned to changes in position and velocity during the movement. On each trial the error between the current motor output in the 2D gain-space and the learning goal, combined with a gradient descent rule, determines how the weights of the respective primitives are updated. This model captures the initially similar motor output in response to the vFF and pFF perturbations, as well as the late-learning rotation of the gain-space trajectory toward the relevant motor learning goal (velocity for vFF training or position for pFF training). However, as mentioned above, this model assumes that the decay of the adaptation is the same for both types of motion-based learning (a symmetric primitive model). This similarity in retention results in a decay trajectory that travels directly back to the baseline value towards the origin. Interestingly, this decay structure makes testable predictions for force-field perturbations that combine velocity- and position-based learning (Fig 4A). First, utilizing the parameters determined from the simultaneous fit to the vFF and pFF behavioral data, for an unbiased combination (ucFF, equally dependent on both motion states) the learning and decay gain-space trajectories will be similar, with the decay closely following the reverse of the adaptation path. Second, using the same model parameters, the decay for adaptation to a position biased force-field (pcFF, a greater position and smaller velocity dependence) will be biased towards the position axis due to the greater representation of position-based learning at the end of training.

In our modification to this model we assume, based on the behavioral results above (see S2 Fig and S3 Fig), that the retention of learning based on changes in movement velocity is greater than the retention of learning based on changes in limb position. That is, during adaptation, the portion of the primitive population that encodes velocity information maintains a larger representation of this motion-based learning. We modeled this asymmetry by imposing that each primitive has two decay rates, one for position-based learning and one for velocity (see Materials and Methods). In this case, on each trial the amount of adaptation is scaled with different non-unity factors for position and velocity. We refer to this implementation as the asymmetric primitive model and, similar to the symmetric model, we applied this model to the vFF and pFF behavioral data and made predictions for force-field perturbations that combine velocity- and position-based learning (see Materials and Methods). When determining the values of the respective retention factors, we did not put any constraint on the relationship. Thus, the retention asymmetry could be in either direction, allowing a direct assessment of any difference.

This asymmetric model makes similar predictions as the symmetric model for the time course of adaptation for pFF and vFF training (Fig 4B). However, only the asymmetric model captures the small, but distinct separation in the decay of pFF and vFF training (see S3 Fig). In addition, the two models make distinct predictions about the decay for ucFF and pcFF training. As described above, the symmetric primitive model predicts equal adaptation to position and velocity, and decay along a similar trajectory for ucFF learning (Fig 4A and 4B). However, the asymmetric model (whose parameters are based solely on the vFF and pFF behavioral data) predicts that the final adaptation to this perturbation is biased toward velocity-based learning, and that the decay lies completely in the portion of the primitive space with more velocity contribution (*α*_*K*_ = 0.942 vs *α*_*B*_ = 0.951). When the two models simulate adaptation to the pcFF perturbation, the symmetric model predicts a decay that remains biased toward position. In contrast, the asymmetric model predicts a shift towards the velocity axis during decay. This is important in the sense that the adaptation endpoint in the primitive gain space imposes distinct decay characteristics under the two models that can directly be tested.

In order to further visualize the differences between the decay trajectories under the two models, we normalized the trajectories with respect to the end point of adaptation (Fig 4C). We did this to remove the effect of the adaptation endpoint for each force-field type, and more importantly reveal the difference between the decay rates. Under both ucFF and pcFF, the symmetric primitive model predicts a decay that follows the unity, x = y line. This is expected due to the same decay rates for position- and velocity-based learning. In contrast, the asymmetric model (whose parameters in this case are based only on the vFF and pFF data) predicts that the decay will be biased towards the velocity axis. The bias in the decay is predicted by the larger retention rate for velocity state compared to position (S6 Fig).

Although both models fit the pFF and vFF data qualitatively, it is important to note that there are aspects of the simulated adaptation that both models fail to capture (e.g., differences in the initial adaptation trajectory (magnitude and direction) between pFF and vFF, Fig 3A and 3B). For additional insight into these differences we focused on the predictions of the asymmetric model simulation, fitting the model separately to the vFF and pFF data in S4 Fig. Note that as in Fig 4, the values of the respective retention factors were not constrained. Consistent with Fig 4, in all cases the normalized decay trajectory is above the unity line demonstrating that velocity-based learning is decaying slower than position-based adaptation. Additionally, in S5 Fig and S6 Fig we show the influence of the primitive distribution on the learning trajectory and the influence of the retention rates on the decay trajectory. Finally, based on the same parameters in Fig 4, we also simulated the decay for adaptation to a velocity biased force-field (vcFF, a greater velocity and smaller position dependence, S7 Fig). Although the learning trajectory mirrors the pcFF simulation, the relative stability of the motion-based adaptation are consistent with Fig 4C.

### Gain-space analysis of training in state-dependent force-fields based on position and velocity

To test the predictions of the symmetric and asymmetric primitive models we trained two additional groups of subjects in force-field perturbations that were unbiased (ucFF) and position biased (pcFF) combinations of the two motion states in order to further characterize the stability of velocity- and position-dependent learning. Previous studies have shown that the adaptation rate to a force-field with a positive correlated dependence on limb position and velocity is faster than adaptation to a purely position or velocity-dependent force-field [16]. Here, we examined how the ratio of position and velocity dependence influenced the motor adaptation and stability during the decay period. As described for the simulations above, we first examined adaptation and decay in response to a force-field equally dependent on both motion states (ucFF). Following this, we examined learning and the subsequent decay for movements made within a combination force-field with a greater position and smaller velocity dependence (pcFF).

**Fig 5:**
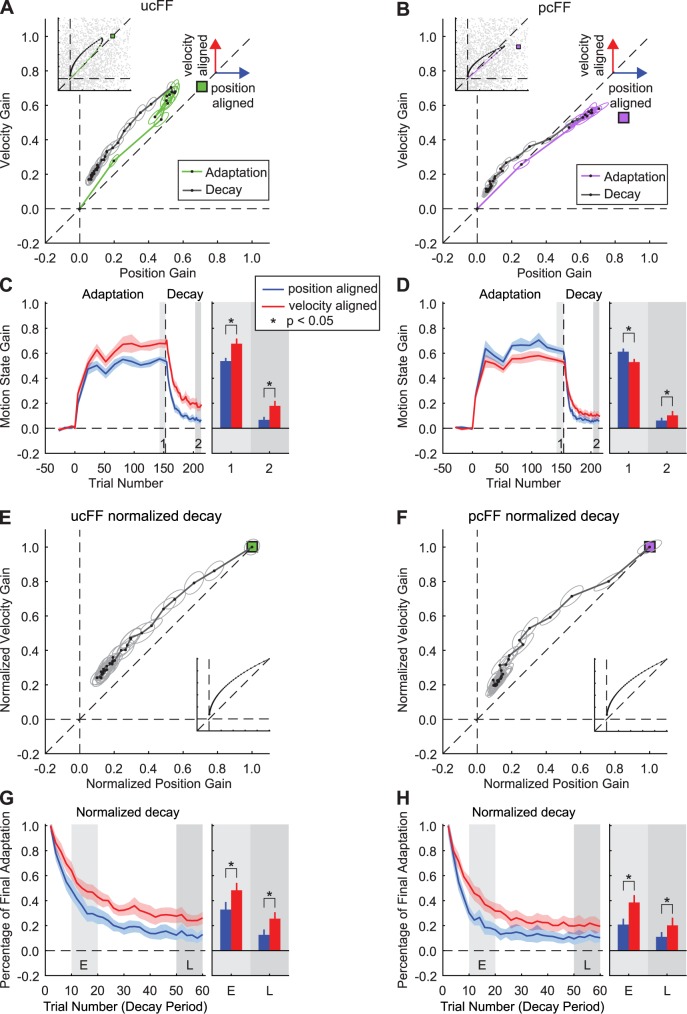
Gain-space representation of adaptation and decay for training in combination force-fields. **(A and B)** Gain-space trajectories during training in the unbiased combination (ucFF, green trace) and position biased force-field (pcFF, purple trace) are shown. The respective gain-space trajectories during the decay periods are shown in gray. The learning goal of the adaptation in gain space is shown by the filled green and purple squares. The directions of position-aligned and velocity-aligned components are shown by blue and red arrows, respectively. **(C and D)** Evolution of the applied position- and velocity-dependent gains during adaptation and decay of the combination force-field training. Position-dependent gains are shown in blue and velocity-dependent gains are shown in red. Each point during the adaptation period is the average across subjects for windows of 10 or 15 trials. The bar graphs show the amplitude of each component for the shaded regions numbered in panels A and B. **(E and F)** The normalized gain-space trajectories during the decay periods were computed by rescaling the gains during decay by their respective values at the start of the decay period. Thus, the first point is rescaled to [1.0, 1.0] in gain space. The gray lines represent the normalized decay in gain space, and the ellipses show standard error at each point. **(G and H)** Normalized decay of position- and velocity-dependent gains. The bar graph depicts the comparison between the normalized gains in the early (E) and late (L) epochs. Error bars show standard error of gains for each epoch. Insets in panels A, B, E and F show the predictions of the asymmetric primitive model fit simultaneously to the ucFF and pcFF behavioral data (*α*_*K*_ = 0.914, *α*_*B*_ = 0.958, *σ*_*K*_ = 0.546, *σ*_*B*_ = 0.565, *η* = 1.5 x 10^−4^, *ρ* = 0.48).

We observed that the adaptation to an unbiased combination force-field (equally dependent on the state of the position and velocity during the movement) was generally closer to the learning goal by the end of the adaptation period (Fig 5A). The applied gain was significantly different between the late periods of adaptation and decay, and between the two types of motion states (2-way ANOVA, *P* < 0.001 for both the main effect of period and the main effect of motion state). Subjects initially adapted to the force-field by applying similar state-dependent gains for changes in position and velocity (position: 0.20 ± 0.03, velocity: 0.28 ± 0.04, *P* = 0.09, paired two-tailed t-test). However, by the end of the adaptation period, the velocity-dependent gain was significantly greater than the position-dependent gain (position: 0.54 ± 0.03, velocity: 0.68 ± 0.04, *P* < 0.05, paired two-tailed t-test). This resulted in a gain-space learning trajectory that was above the unity line and clearly biased towards the velocity-dependent gain axis (ordinate in Fig 5A).

To examine the characteristics of this bias, we projected the gain-space trajectory onto the position and velocity gain axes at each point during learning and decay (Fig 5C). As described above, early in adaptation the position- and velocity-dependent gains had similar magnitudes, but the velocity-dependent gain was significantly greater than the position-dependent gain by the end of the adaptation period. This significant difference between the velocity- and position-dependent gains extended throughout the decay period (Late in decay: position: 0.07 ± 0.02 compared to velocity: 0.18 ± 0.03, *P* < 0.05 for both cases, paired two-tailed t-test, Fig 5C bar graph). An exponential fit to the decay of the velocity and position components showed a larger time constant for velocity (time constant of 9.4 ± 1.8 trials for position compared to 12.1 ± 2.0 trials for velocity. See S8A Fig). Additionally, the applied gains based on velocity and position at the end of the decay period remained significantly greater than baseline levels (velocity: 3.8 x 10^−4^ ± 0.007 vs. 0.18 ± 0.03, position: 0.003 ± 0.007 vs 0.07 ± 0.02, paired two tailed t-test, *P* < 0.001 for both cases). This clearly shows that when the force-field is equally dependent on changes in movement position and velocity, the gain of the velocity-dependent force contributed more in both the adaptation and decay periods. This is in agreement with the predictions of asymmetric primitive models, which suggest a bias late in adaptation toward velocity continuing throughout the decay period (Fig 4B).

One might suspect that the observed bias in the decay trajectory for the unbiased combination force-field is the consequence of the unbalanced adaptation levels; the final adaptation has a significantly greater velocity-dependent gain compared to position. In order to remove this confound, we normalized the gain-space trajectory during the decay by the position and velocity-dependent gains by the respective values at the beginning of the decay period. Thus, the rescaled initial point of decay in gain-space is located at [1.0, 1.0]. If the shape of the decay gain-space trajectory in Fig 5A was the result of unequal learning at the end of adaptation, then the normalized decay should be aligned with the equality line in gain space. However, the normalized trajectory clearly shows that the velocity-dependent gain was always greater than the respective position-dependent gain throughout the decay period (Fig 5E). When we examined the temporal changes of the normalized gains during decay (Fig 5E) by projecting the trajectory onto the position and velocity-dependent gain axes, we observed the same effect (Fig 5G). The percentage of adaptation at the beginning of the decay period was significantly greater than at the end, and the percentage of adaptation based on velocity and position was significantly different (2-way ANOVA, *P* < 0.001 for the main effect of period and the main effect of motion-based learning). For the early and late epochs of decay, the normalized velocity-dependent gain was significantly greater than position (early epoch: position: 37 ± 7% vs. velocity: 52 ± 6%; Late epoch: position: 12 ± 5% vs. velocity: 25 ± 5%; *P* < 0.05 for all cases, paired two-tailed t-test). This is in line with the decay predicted by the asymmetric primitive model and suggests that there is an asymmetry in the retention rates between position- and velocity-based motion-state learning (Fig 4C).

As stated previously, a potential confound for the unbiased combination force-field is that the velocity-dependent gain at the end of adaptation was significantly greater than position. This may have influenced the decay and resulted in the velocity contribution being more stable throughout the decay period. We therefore conducted an additional experiment using a position-biased combination force-field (pcFF). As predicted by both models, at the end of training the adaptation gain-space trajectory for this force-field is biased toward the position axis (abscissa) as shown in Fig 4A and 4B. However, as the decay period starts, the asymmetric model predicts that the gain-space trajectory will move toward the velocity-dependent gain axis (ordinate) and remain above the unity line for the remainder of the decay period (Fig 4B). In contrast, the symmetric model predicts that the adaptation will decay towards the position axis (Fig 4A)

Fig 5B shows the behavioral results for subjects trained on this combination force field. The decay of adaptation is clearly biased towards the velocity axis, consistent with the predictions of the asymmetric model. This can be seen in the trial-by-trial changes of both gains during adaptation and decay period (Fig 5D). The applied gain was significantly different between the late periods of adaptation and decay, but there was no main effect of motion state (2-way ANOVA, *P* < 0.001 for the main effect of period and *P* = 0.27 for the main effect of motion state). (Note that the non-significant effect of motion state is due to significant effects in opposite directions in the late periods of adaptation and decay. See below.) Similar to the ucFF results, an exponential fit to the decay of the velocity and position components showed a larger time constant for velocity (time constant of 5.7 ± 0.6 trials for position compared to 9.3 ± 0.9 trials for velocity. See S8B Fig). In addition, the final gains applied to velocity and position at the end of the decay period remained significantly greater than baseline levels (velocity: 0.001 ± 0.007 vs. 0.10 ± 0.03, position: 8.9 x10^-4^ ± 0.007 vs. 0.06 ± 0.02, paired two tailed t-test, *P* < 0.001 for both cases). Although the adaptation starts with equal contribution of both motion components, late adaptation is significantly dominated by the position-dependent learning (Early adaptation: position: 0.26 ± 0.05 vs. velocity: 0.26 ± 0.03, *P* = 0.99, paired two-tailed t-test; Late adaptation: position: 0.61 ± 0.03 vs. velocity: 0.53 ± 0.02, *P* < 0.05, paired two-tailed t-test). At the start of the decay period there is a rapid drop in the position-dependent gain. However, the decay of the velocity-dependent gain is much slower, resulting in the gain-space trajectory remaining above the unity line throughout much of the decay period (Late in decay: position: 0.06 ± 0.02 vs. velocity: 0.10 ± 0.03, *P* < 0.05, multiple comparisons corrected).

Due to the significant difference in the gain magnitudes at the end of the training, we also examined the normalized decay for pcFF training. Similar to the results for the unbiased combination force-field, we observed that the normalized decay gain-space trajectory was above the unity line for the entire decay period, indicating that the velocity-dependent gain decayed at a slower rate than the position-dependent gain. In addition, the percentage of adaptation at the beginning of the decay period was significantly greater than at the end, and the percentage of adaptation based on velocity and position was significantly different (2-way ANOVA, *P* < 0.001 for the main effect of period and the main effect of motion-based learning). This effect is strongly present in both early and late epochs of the decay period (Early, position: 24 ± 5% vs. velocity: 42 ± 6%; Late position: 11 ± 4% vs. velocity: 20 ± 6%; *P* < 0.05 for both cases, paired two-tailed t-test) (Fig 5F and 5H). This again is in agreement with the simulations from the asymmetric primitive model (Fig 4B and 4C, and insets in Fig 5A, 5B, 5E and 5F). When this model was applied simultaneously to the ucFF and pcFF behavioral data the simulations (insets in Fig 5A, 5B, 5E and 5F) predicted adaptation during training to be biased toward the position axis, but with the start of the decay, the gain-space trajectory is biased towards the velocity axis due to an asymmetry in the stability of the motion-state learning (*α*_*K*_ = 0.9424 vs *α*_*B*_ = 0.9654).

## Discussion

We designed a series of experiments to examine the stability of motion-state based updates to motor commands in response to the introduction of novel dynamics during reaching movements. In our first experiments these dynamics were dependent either solely on changes in movement position or velocity. We directly measured the temporal force patterns subjects applied via error clamp trials, during which the robotic manipulandum constrained the movement to a straight trajectory between targets by counteracting any perpendicular motion. Based on the force patterns subjects applied to counter the perturbation, we determined the gain associated to changes in movement position and velocity. We determined the applied gains in response to different types of dynamics and examined the stability of these modifications to motor commands when the perturbation was removed and subjects only made error clamp movements. When the respective gains were represented in a two-dimension gain space we observed a separation between the gain-space trajectories during the learning and the decay periods. Based on the observed behavioral differences in the retention of the learning between pFF and vFF training, we modified a previous model of motor adaptation and made several predictions on the decay of learning following training in force-field perturbations that were a combination of both motion states. Interestingly, the simulations predicted that when the learning goal had partial dependence on both motion states the position-dependent gain would decay at a faster rate relative to the velocity-dependent gain. This was the case even when the gain associated to position at the end of training was significantly greater than that applied to movement velocity. These simulations were confirmed by a second set of experiments in which we examined the learning and decay to these combination motion-state perturbations. Together, our simulation and behavioral results suggest that (1) overlapping, but distinct processes underlie motor adaptation and its decay and (2) the adjustment of motor commands based on movement velocity is relatively more stable than that based on position.

### Different processes underlie motor learning and decay

The gradual decay of newly formed motor memories has been studied for different contexts and tasks, including: prism [6,7], locomotion [8,9], visuomotor [10,11] and force-field perturbations [4,12,15]. The decay of adaptation in these studies is often at a different rate compared to initial learning suggesting at least partially separate mechanisms [23,27]. Recently, Kitago and colleagues [5] examined the decay of visuomotor adaptation for different types of assessments. For all methods examined, there was a decay in the adaptation, but the rate was the fastest when the perturbation was removed and slowest when the errors orthogonal to the ideal movement trajectory were visually clamped to zero (similar to the error clamps used in the current study). (Note the minimum decrease in adaptation level occurred with the passage of time, but the decay rate is difficult to quantify and compare for this context.) This suggests that the method in which the decay rate is assessed (i.e., the context of the decay period) potentially has a considerable influence on the decay rate of the motor memory [13,14,28].

In order to evaluate modifications to the feedforward changes in the motor output, it was necessary to utilize error-clamp trials. Although it is possible that assessing adaptation decay in a different manner (e.g., decay trials with perturbation removal) could influence the reductions in motor output we report, our main interest was how these changes in motor output compared to the initial learning and varied for different learning goals. Our results show clear separation in the applied gains to changes in position and velocity between the initial learning of the novel dynamics and the subsequent decay of the motor adaptation. In all cases, the applied gain did not return to baseline levels. This was true for the goal aligned motion state (Fig 3C and 3D) when the perturbation was based on a single state, and when the perturbation had a codependence on both motion sates (Fig 5C and 5D). This behavioral difference mirrors the neuronal retention of learning reported throughout the sensorimotor system following motor adaptation. The activity of a population of premotor, supplementary motor, and primary motor cortex cells is modified during force-field adaptation and these correlated modifications are retained during the decay of the learning, serving as a memory trace of the training [29–33]. The behavioral difference between learning and decay we report may reflect these persistent neural changes throughout the sensorimotor system specifically tuned to the motion kinematics required for force-field compensation.

Although the focus in our study was the examination of the decay of the velocity- and position-dependent learning, there are some aspects of the adaptation trajectories that are not captured by the primitive model (e.g., differences in the initial adaptation trajectories in Figs 3 and 5, compared to the simulations in Fig 4). These inconsistencies may be due to several interesting factors resulting from perturbation-dependent changes in the primitive distribution. The supplemental simulations (S5 Fig and S6 Fig) suggest that there are possible changes in the primitive distribution (at least within this computational framework) occurring during the two types of training (vFF and pFF) that influence the learning trajectories; it is possible that the primitive space may rotate during training, but the extent towards a particular axis may have different rates. We plan to address this possibility with future, systematic experiments.

Finally, the sensory adaptation that occurs with motor learning may provide an additional measure to assess differences in adaptation retention. Ostry and colleagues [34] demonstrated that following the exposure to a force-field movement perturbation there was an accompanying modification in the perception of limb position. Specifically, the perceptual shift was in the direction of the movement disturbance and learning-dependent; there was no observed sensory modification when the limb was moved passively through the same trajectories experienced during the motor perturbation. Thus, another possible assessment of any asymmetry in the retention of the velocity and position components could be to compare the magnitude of the accompanying perceptual shifts in limb estimation and the degree to which these perceptual modifications persists throughout the decay period.

### Influences on the stability of motor learning

Several studies have suggested that how the perturbation is introduced (e.g., abruptly vs. gradually) and the duration of exposure (e.g., long vs. short) influence the stability and subsequent properties (transfer, long-term retention, etc.) of motor adaptation [21,35–37]. For example, Huang and Shadmehr [22] showed that when the force-field perturbation was applied for a short duration, the decay of the adaptation was much more rapid than for longer training periods, suggesting less relative stability in the modifications to the motor commands. This is in agreement with recordings in motor cortex [38]; the activity of a subset of neurons is modified dependent on the rate of movement perturbations experienced, indicating that the neural representation of adaptation is influenced by the training schedule.

In addition to training schedule, previous studies have examined factors that influence the stability of adaptation retention [12,17–21,39,40]. However, an important distinction of the current study is that we examined the stability of the *components* of the motor adaptation (the motion-state based learning) rather than the long-term stability of the adaptation or stability in competition with the formation of other motor memories. As in Sing et al. [16], our current observations show that the motor memory in the late stages of training is more specific to the task goal compared to the initial stages due to modification in the gains applied to the goal-aligned and goal-misaligned motion parameters. This specificity in the motor output remains throughout the decay period; there is no reemergence of the initial goal-misaligned learning as the acquired goal-aligned learning gradually decays. Based on the collective work described above, it would be interesting to examine the influence of the (1) training duration, (2) introduction rate and (3) passage of time on the stability of these adjustments to the motion state gains. For example, there is recent evidence that performance becomes more task specific with sufficient breaks after training, suggesting that the passage of time may influence the ability to perform more task-relevant actions [41]. We hypothesize that the well-known savings following a break after initial training (faster re-adaptation with exposure) will reflect more goal-aligned movements [10]. That is, savings over multiple days of training should result in adaptation gain-space trajectories closer to the goal-aligned axis (the motion kinematic of the experienced force-field) than on the first day of exposure.

### Physiological implications of the greater relative stability of velocity-dependent learning

As demonstrated previously [16,24,25], the initial adaptive responses that we observe when learning novel movement dynamics are consistent with motor primitives with correlated position and velocity tuning. This theoretical framework is based on the codependent encoding of these motion states observed throughout the sensorimotor system [42–47]. Our current results suggest that this codependence does not necessarily result in an equal representation of the two motion states, but rather codependent processing biased towards velocity. For example, similar to previous studies [16,24,25], initial position-dependent learning is biased towards the velocity-dependent gain; the gain-space trajectory is typically closer to the middle of the gain space than that observed for velocity-dependent learning (Fig 3A and 3B). In addition to initial learning, the decay of the position-dependent gain was relatively faster than the reduction of the velocity-dependent gain for both combination force-fields, suggesting an asymmetry in relative stability (Fig 5).

Why should learning based on movement velocity be more stable than that based on position? A possible answer may be found in the encoding asymmetries in motor cortex [47]. Velocity tuning among primary motor cortex neurons is more abundant compared to position. Another reason for a velocity bias could be that movement velocity provides substantially more motion information compared to position. For example, during point-to-point movements, there can be a significantly larger variance in the temporal changes in movement velocity for similar movement trajectories [48]. Take for example the force-field perturbations used here; the peak force experienced by the subject can vary broadly when based on movement velocity, whereas this peak is restricted when based on positional changes. If such a coding bias exists throughout the sensorimotor system, this imbalance would support a preference towards velocity-based learning during initial force-field adaptation and an asymmetry during the subsequent decay. Possible support for this bias may be found in a recent study by Rotella and colleagues [49]. The authors asked subjects to produce isometric hand forces which were then mapped to the position or velocity of a virtual cursor. Under these different mappings, they then tested the generalization of adaptation when a visuomotor rotation was applied to the cursor motion. Interestingly, the generalization of adaptation under the velocity mapping was broader, which is aligned with the current implications that movement velocity is a more stable basis for motor learning than changes in position.

### Conclusion

We investigated the decay of short-term adaptation to motion-dependent perturbations applied to reaching movements. We observed a clear separation between the initial learning and subsequent decay when the motor output was represented as the respective gains subjects applied to changes in position or velocity during movement. When the perturbation was only based on one motion state (position or velocity), this separation was a direct effect of a sustained decrease in the gain of the goal-aligned motion parameter, with no reemergence of the goal-misaligned parameter during the decay period. When exposed to novel dynamics that required a combination of position- and velocity-dependent learning, the applied velocity-dependent gain was relatively more stable during the decay period, even when the gain applied to changes in position was significantly greater at the end of training. This difference in the relative state-dependent learning stability suggests that the motor system has an inherent preference towards adjusting and retaining modifications to motor commands based on movement velocity. A modified model of adaptation that accounts for greater retention of velocity-based learning captures these behavioral results, and importantly predicts the decay behavior for training with novel force-fields that are jointly dependent on the two motion states. Overall our results show that the decay of motor adaptation is not exactly *unlearning*—the complete reversal of the learning process. Rather, in agreement with previous physiological and behavioral studies, our results suggest that the decay of adaptation likely shares overlapping mechanisms with the learning process, but is a distinct process that reduces the motor memory traces formed over the training period.

## Materials and methods

### Ethics statement

The study protocol was approved by the George Mason University Institutional Review Board, and all participants gave informed written consent.

### Participants

Fifty-six healthy subjects (37 male and 19 female) without known neurological impairment were recruited from the George Mason University community to participate in the study. All participants were right-handed and performed the task using their right hand. Each individual participated in only one of the experimental sessions and experienced only one type of force-field (14—Velocity-dependent Force-field, 14—Position-dependent Force-field, 14—Unbiased Combination Force-field, and 14—Position-biased Combination Force-field).

### Experimental setup

The experimental paradigm was based on the standard force-field adaptation paradigm [26]. The subjects were instructed to move a cursor between two targets located on a screen in the sagittal axis of their body while grasping a robot manipulandum (KINARM End-Point Lab, Fig 1A). The manipulandum measured hand position, velocity, and the force applied by subjects, and its motors were used to apply forces to the hand, all at a sampling rate of 1000 Hz. A semi-transparent mirror was used to project the location of hand and visual targets to the plane of movement while occluding the subject’s view of the hand (refresh rate of 60Hz). During the experiment the subjects reached to circular targets 0.6 cm in diameter that were spaced 10 cm apart on the sagittal axis of the body. The subjects were instructed to ‘‘make quick reaching movements to the targets in both the forward and backward directions.” At the end of each trial, subjects received visual and auditory feedback about the completed movement. If the peak movement velocity was between 0.25–0.35 *m*/*s* and the movement duration was shorter than 750 ms, the reach target (green target in Fig 1A) filled green with an auditory reward indicating a movement within the required criteria. If the peak movement speed was below 0.25 *m*/*s*, the reach target filled yellow to indicate that the movement was too slow. If the peak movement speed was above 0.35 *m*/*s*, the reach target filled red to indicate the movement was too fast. In both of the latter cases no auditory feedback was given. The endpoint of each movement was used as the start point for the following trial, and movements were made only in these two directions. The subjects received a performance score at the end of each block of movements that indicated the percentage of correct trials only in the trained 270° movement direction. Subjects were asked to maintain the score above 50% throughout the experiment. Only 270° movements with a peak velocity between 0.2–0.4 *m*/*s* were used in the subsequent data analysis. In addition, subjects had to initiate their movement within 75–2000 ms after the reach target appeared on the screen. Otherwise all targets were extinguished and the trial was immediately repeated.

Three trial types were used during the experiment: null trials, force-field (FF) trials, and error-clamp (EC) trials (Fig 1B). Null trials were used for initial practice, during which the motors of the robot manipulandum did not apply any force to the hand. During FF trials, the robot applied a force at the hand that was dependent either on movement position (with respect to the start location), velocity, or a positive combination of limb position and velocity. The force that the robot applied to the hand was always orthogonal to the direction of movement, and had the general form of:

$$\left[\begin{array}{c} F_{x}\\ F_{y} \end{array}\right]=c_{K}.\left[\begin{array}{cc} 0 & -K\\ K & 0 \end{array}\right].\left[\begin{array}{c} x\\ y \end{array}\right]+c_{B}.\left[\begin{array}{cc} 0 & -B\\ B & 0 \end{array}\right].\left[\begin{array}{c} \dot{x}\\ \dot{y} \end{array}\right],\;K=45\frac{N.s}{m},\;B=15\frac{N}{m}$$ (1)

For a position-dependent force-field trial (pFF), *c*_*K*_ = ±1 and *c*_*B*_ = 0, where *c*_*K*_ = ±1 and *c*_*K*_ = −1 correspond to clockwise and counterclockwise direction of the force-field, respectively (a clockwise force-field is shown in Fig 1B). For a velocity-dependent force-field trial (vFF), *c*_*K*_ = 0 and *c*_*B*_ = ±1. Unbiased combination force-field trials (ucFF) had a force pattern dependent on both the position and velocity, with *c*_*K*_ = ±0.71 and *c*_*B*_ = ±0.71 for clockwise and counterclockwise directions [16,23,24]. Lastly, the Position-biased combination force-field trials (pcFF) had a motion dependent force pattern similar to the ucFF. However, the contribution of the position-dependent component was 20% greater and the velocity-dependent component was 25% less, with *c*_*K*_ = ±0.85 and *c*_*B*_ = ±0.53. As in Sing et al. [16] the values for *K* and *B* were chosen in order to have approximately equal peak perturbing force for vFF and pFF. Each subject experienced only one type of force-field throughout the experimental session.

During error-clamp trials, the robot motors constrained movements in a straight line toward the reach target by counteracting any motion perpendicular to the target direction [21,50]. This was achieved by applying a stiff one-dimensional spring (6 kN/m) and a damper (150 Ns/m) in the axis perpendicular to the reach direction. In these trials, perpendicular displacement from a straight line to the reach target was held to less than 0.6 mm and averaged about 0.2 mm in magnitude.

### Task

Each subject experienced the same basic experimental paradigm shown in Fig 1C. Subjects performed sets of 90° and 270° movements. Each experiment started with a baseline period, during which subjects completed 360 null trials (180 movements in the trained 270° direction). These null trials were divided into 4 blocks. The first two blocks had 80 movements each and the last two blocks each required 100 movements. During the last 2 blocks of trials 12 error-clamp trials were pseudo-randomly interspersed for the 270° movement direction in order to measure the baseline levels of forces for each subject. The average lateral forces during these trials were then subtracted from the forces applied on error-clamp trials during the adaptation and decay periods.

Following the baseline period, subjects experienced the adaptation transition block (124 total movements, 62 in the trained 270° direction), during which the force-field environment was suddenly introduced after an initial 30 null movement trials (15 in the trained 270° direction). We designed the adaptation transition block to capture the immediate changes in the applied force due to initial exposure to the force-field. Once the perturbation was introduced all 90° movements were made under the error-clamp condition, and the force-field was only applied to 270° movements. For the first 10 training trials, the ratio of force-field (FF) to error clamp (EC) trials was 3 FF: 2 EC which was then reduced to 5 FF: 1 EC for the last 84 trials (42 in the trained direction). The adaptation transition period was followed by 2 blocks of training (96 total trials each) in which the subjects experienced only one of the four force-field environments (vFF, pFF, ucFF, or pcFF). Similar to baseline period, we pseudo-randomly inserted 16 error-clamp trials in the 270° movement direction in order to measure the adaptation level at different points in training. The ratio of 5 FF: 1 EC was maintained throughout this training period. Only the 270° direction movements were used for analysis of adaptation and decay. The sign (direction) of the FF remained constant for each subject, but was counterbalanced between subjects.

After the adaptation period, subjects experienced the decay transition block of 146 total trials. This block started with 26 training trials. For the first 12 trials, there was a ratio of 5 FF: 1 EC which increased to 4 FF: 3 EC for the last 14 trials in order to obtain an accurate measure of final adaptation levels. These 26 trials were then followed by 120 consecutive error-clamp trials (60 in the trained 270° direction). We refer to these 120 error-clamp trials as the decay period, during which the adaptation decayed to the baseline levels prior to experiencing the force-field. Inclusion of the decay period within the transition block effectively masked any possible context dependent changes in the behavior of the subject due to the removal of the force-field [14,28]. We used 60 consecutive error clamp trials to measure adaptation decay in order to keep this critical experimental block within a reasonable duration, and avoid breaks and possible cognitive influences during the transition to the decay period. In addition, based on the exponential decay time constants (S1 Fig, S2 Fig, S8 Fig), this number of error clamp trials proved sufficient to observe asymptotic levels of decay.

### Analysis of force profiles

As described previously [16,21,37,50,51] we used error-clamp trials to measure the change in feedforward motor output during the adaptation and decay periods. The use of error-clamp trials reduces the lateral errors experienced during the movement that elicit online feedback correction. Given that the lateral force during error-clamp trials reflects the predictive feedforward adaptive response to the force-fields, we limited our analysis to these force patterns. Based on Eq 1, subjects fully compensate for the force-field when they produce a countering force that is proportional to the movement velocity, position, or the positive combination of the two. We first computed the ideal force pattern by examining the longitudinal movement kinematics (position, velocity) during the error-clamp trial movement. The movement and force signals were analyzed within a temporal window of 1500 ms centered on the peak velocity (±750). Next we defined the adaptation coefficient by determining the linear regression coefficient between the ideal force and the lateral force applied by the subject during the error-clamp trials [16,21,37,50,52]. We computed the adaptation coefficient for each subject during both the adaptation and decay periods and averaged the values over all subjects. In all cases we provide the SEM of this average value.

We further characterized the adaptation and decay behavior by projecting the lateral force during each error-clamp trial onto a two-dimensional space that parsed the position-dependent and velocity-dependent components of the applied force [16]. We refer to this two-dimensional space as the gain-space. This gain-space represents complete adaptation to a vFF by the point [0,1], pFF by the point [1,0], ucFF by the point [0.71, 0.71], and a pcFF by the point [0.85, 0.53]. Additionally, the abscissa and ordinate of each point in this gain-space corresponds to the position-dependent and velocity-dependent components of the applied force. In order to depict adaptation and decay in gain-space, we first calculated a multiple regression between the lateral force during the error-clamp trials, and both the changes in position and velocity during the movement. We then rescaled the coefficients for the position and velocity components by the 45N/m and 15Ns/m factors, respectively, and projected these coefficients onto the gain-space. For each subject, we performed this analysis and calculated the average gains over all subjects [16,23–25].

Similar to Sing et al. [16], the characterization of position and velocity contributions in the force output produce excellent fits (R^2^ values ranging from 0.91 to 0.99, see Fig 2). As in this prior study, the inclusion of an acceleration term resulted in highly significant but relatively small improvements in the representation of these force profiles; in the majority of cases for the different types of perturbations (vFF, pFF, ucFF, and pcFF; early and late) the acceleration signal’s contribution was significant (*P* < 0.001 in all cases except early pFF training, *P* = 0.38), but the overall force profile variance accounted for only improved by at most 3%. We therefore elected to focus only on the contributions of the position and velocity state variables.

We operationally defined early and late/asymptotic adaptation as the first 15 (1–15) and last 10 (150–160) trials of training. Thus, the mean and standard error values for these periods are plotted as a function of the mean trial number within these windows for the adaptation period (Figs 2, 3 and 5). The data during the decay periods were normalized by dividing all the subject data by the mean (across subjects) of the first decay trial. Due to the increased frequency of EC trials during the decay period, we used a smaller window to assess early and late levels (trials 11–20 and 50–60 respectively). Here we excluded the first 10 trials in the analysis for the early epoch in order to remove the effect of normalization of adaptation gains (Fig 5G and 5H). We initially tested the main effect of group condition on the different epochs of interest with a repeated measures ANOVA and subsequently determined the epoch in which these conditions were significantly different with post-hoc analysis. For example, two-tailed t-tests were performed between different force-field groups to compare the behavior within each epoch. For all tests the significance level was 0.05.

### Exponential fits

In S1 Fig, S2 Fig and S8 Fig we applied a standard exponential model with rate and offset parameters to determine the time constants of learning and decay for the different types of perturbations (vFF, pFF, ucFF and pcFF) and learning components (goal-aligned and goal misaligned, velocity- and position-based). We computed the standard deviation of the best-fit parameter values for these model fits by bootstrapping the fits to the data. We made 500 different bootstrap estimates of the fit parameters, each by averaging data from 14 randomly generated choices made from the 14 subject data pool with replacement. We fit the model to each of these bootstrap estimates and determined the standard deviation of each parameter.

### Symmetric and asymmetric viscoelastic primitive model

The viscoelastic primitive model first proposed by Sing et al. [16] consists of *N* motor primitives, *S*_*i*_ = [*K*_*i*_ *B*_*i*_]^*T*^ = *R*^*n*×2^, which collectively generate the motor output. The primitives are jointly distributed as

$$[\begin{array}{cc} K_{i} & B_{i} \end{array}]\sim N(\mu ,\Sigma ),$$

$$\mu =[\begin{array}{cc} 0 & 0 \end{array}];$$

$$\Sigma =\left[\begin{array}{cc} \sigma_{K}^{2} & \rho \sigma_{K}\sigma_{B}\\ \rho \sigma_{K}\sigma_{B} & \sigma_{B}^{2} \end{array}\right]$$

In this model these primitives have a similar dependency on position and velocity via *σ*_*K*_ = *σ*_*B*_. Moreover, the correlation between the primitives is determined by *ρ*.

The motor output on each trial is determined by a weighted combination of motor primitives. Each primitive receives input from the changes in position and velocity during the movement and creates a force output:

$$F_{S_{i}}={\left[\begin{array}{c} K_{i}\\ B_{i} \end{array}\right]}^{T}\left[\begin{array}{c} P\\ V \end{array}\right]$$

Given that the vector [*P V*]^*T*^ is shared between all primitives, we can factor out this vector and simplify the calculation. The final force is a weighted linear combination of the primitive forces:

$$F_{output}=\sum_{i=1}^{n}{w_{i}F}_{S_{i}}$$

$${\left[\begin{array}{c} K_{output}\\ B_{ouput} \end{array}\right]}^{T}\left[\begin{array}{c} P\\ V \end{array}\right]=\sum_{i=1}^{n}w_{i}{\left[\begin{array}{c} K_{i}\\ B_{i} \end{array}\right]}^{T}[\begin{array}{c} P\\ V \end{array}]$$

$$\left[\begin{array}{c} K_{output}\\ B_{ouput} \end{array}\right]=\sum_{i=1}^{n}w_{i}{\left[\begin{array}{c} K_{i}\\ B_{i} \end{array}\right]}^{T}=S^{T}W$$

In this equation the *W* ∈ *R*^*n*×1^ is a weight vector that drives the learning in the model. The output vector [*K*_*output*_ *B*_*output*_]^*T*^ represents the gain in position-velocity (p-v) primitive gain-space, which we refer to as *y*, or the current motor adaptation state. The goal of adaptation can be defined as a vector y^*^ ∈ *R*^2×1^. On each trial of adaptation we can project the error vector between the goal and the motor output and use a gradient descent rule to compute the weight change for each primitive *S*_*i*_ as

$$dw_{i}^{n}=\eta S_{i}(y^{*}-y^{n-1})$$

The weight change can be used to create a new gain state in the p-v primitive gain-space *y*^*n*+1^

$$y^{n+1}=\left[\begin{array}{cc} \alpha_{K} & 0\\ 0 & \alpha_{B} \end{array}\right]y^{n}+S^{T}dW$$

For the symmetric model, the retention value for *α*_*K*_ and *α*_*B*_ are the same. When we applied this model to the vFF and pFF behavioral data (Fig 3) we estimated these parameters to be: *α*_*K*_ = *α*_*B*_ = 0.951, *σ*_*K*_ = *σ*_*B*_ = 0.401, *η* = 1.5 x 10^−4^, *ρ* = 0.51. As with the exponential fits, we made 500 different bootstrap estimates of the fit parameters, each by averaging data from 14 randomly generated choices made from the 14 subject data pool with replacement.

For the asymmetric model, the retention values were not constrained and can result in an asymmetry in either direction. When we applied this model to the vFF and pFF behavioral data we estimated these parameters to be: *α*_*K*_ = 0.942, *α*_*B*_ = 0.951, *σ*_*K*_ = 0.464, *σ*_*B*_ = 0.379, *η* = 1.5 x 10^−4^, *ρ* = 0.47. Note that the retention is biased towards velocity primitives, *α*_*K*_ *< α*_*B*_. In addition, when asymmetric model is applied simultaneously to the ucFF and pcFF behavioral data these parameters were estimated to be: *α*_*K*_ = 0.914, *α*_*B*_ = 0.958, *σ*_*K*_ = 0.546, *σ*_*B*_ = 0.565, *η* = 1.5 x 10^−4^, *ρ* = 0.48.

## Supporting information

S1 Fig Exponential fits to vFF and pFF adaptation and decay. **(A)** A standard exponential model with rate and offset parameters was fit to the trial-by-trial increase in the adaptation coefficient during vFF and pFF training (data presented in Fig 2A). A time constant of 7.2 ± 0.8 was determined for pFF training, and a time constant of 12.3 ± 5.5 trials was estimated for vFF training (mean ± the standard deviation from bootstrapping. See Materials and Methods). **(B)** The exponential model was fit to the decay of the adaptation coefficient following vFF and pFF training. The time constant for decay following pFF training was 11.0 ± 2.2 trials and a constant of 7.1 ± 1.0 trials was determined for vFF training.
(EPS)

S2 Fig Exponential fits to motion-based components of vFF and pFF adaptation decay. **(A)** A standard exponential model with rate and offset parameters was fit to the decay of the goal-aligned component for vFF and pFF training (data presented in Fig 3C and 3D). A time constant of 10.4 ± 2.2 trials was estimated for pFF training and a constant of 7.9 ± 0.9 trials was determined for vFF training. As in S1 Fig, values represent the mean ± the standard deviation from bootstrapping. **(B)** The exponential fit was not a correct representation of the decay of the goal misaligned component. Instead we compared the amount of this component early (trials 10–20) and late (trials 50–60) in the decay period (Early: pFF = 0.08 ± 0.02 vs vFF = -0.02 ± 0.02. Late: pFF = 0.03 ± 0.02 vs vFF = 0.002 ± 0.01). The amount of the goal-misaligned component at the end of the decay period was not significantly different than at the beginning, but the amount of this component was significantly different between perturbation types (2-way ANOVA, *P* = 0.12 for the main effect of decay period, *P* < 0.01 for the main effect of perturbation type). There was a significant difference in the amount of the goal-misaligned component between vFF and pFF training both early and late in the decay period (*P* < 0.05, Bonferroni correction). Thus, there is a significantly larger contribution of the velocity component during the decay of pFF adaptation that decreases at a much slower rate compared to the position contribution to the decay of vFF adaptation.
(EPS)

S3 Fig Simulation of decay for vFF and pFF training for the symmetric and asymmetric viscoelastic primitive models. **(A)** Decay of adaptation for both pFF and vFF training depicted in the primitive gain-space. The abscissa represents the goal-aligned component of learning and the ordinate represents the goal-misaligned component. The gain-space trajectories are rotated such that the learning goal for both types of training are the same in gain-space. For example, the abscissa represents the velocity gain for vFF training and the position gain for pFF learning. The decay for vFF training (red trace) is closer to the goal-aligned axis when compared to pFF training (blue trace). Prediction of the decay gain-space trajectories for pFF and vFF training for the **(B)** symmetric and **(C)** asymmetric primitive viscoelastic models. In both cases the model parameters are based on fits to the vFF and pFF behavioral data (Fig 3). The panels are the same representation as in panel A. Although small, the asymmetric model’s prediction of the trajectory separation for the decay of vFF and pFF training are in agreement with behavioral results in panel A.
(EPS)

S4 Fig Simulation of the asymmetric viscoelastic primitive model based on separated data sets. Simulations of the learning and decay for adaptation to ucFF and pcFF perturbations when the asymmetric model was separately fit to the **(A)** pFF (*α*_*K*_ = 0.951, *α*_*B*_ = 0.971, *σ*_*K*_ *= σ*_*B*_ = 0.40, *η* = 1.5 x 10^−4^, *ρ* = 0.45) and **(B)** vFF data (*α*_*K*_ = 0.885, *α*_*B*_ = 0.936, *σ*_*K*_ *= σ*_*B*_ = 0.4, *η* = 1.5 x 10^−4^, *ρ* = 0.52). In these fits the primitive variances were constrained to be equal to each other in order to limit the number of free parameters estimated from the reduced data set. However, the retention values were not constrained and could result in an asymmetry in either direction. Adaptation is represented by the colored traces, and the black traces represent decay. The second row in each panel shows the normalized decay of position- and velocity-dependent gains for the respective perturbation. In all cases the decay of the velocity-based learning is slower than position-based; the normalized trajectory is above the unity line demonstrating that velocity-based learning is decaying slower than position-based.
(EPS)

S5 Fig The influence of the covariance matrix on simulated learning trajectories. The simulation of the asymmetric model for the learning (colored traces) and decay trajectories (black traces) in primitive gain space for different correlations between position and velocity primitives: symmetric distributions **(A)** (*α*_*K*_ = *α*_*B*_ = 0.98, *σ*_*K*_ = *σ*_*B*_ = 0.5, *η* = 1.5 x 10^−4^, *ρ* = 0.8), distributions skewed towards the position axis **(B)** (*α*_*K*_ = *α*_*B*_ = 0.98, *σ*_*K*_ = 0.5, *σ*_*B*_ = 0.4, *η* = 1.53 x 10^−4^, *ρ* = 0.52) and distributions skewed towards the velocity axis **(C)** (*α*_*K*_ = *α*_*B*_ = 0.98, *σ*_*K*_ = 0.4, *σ*_*B*_ = 0.5, *η* = 1.5 x 10^−4^, *ρ* = 0.8). In each case, the retention rates for velocity- and position-based learning, and the correlation are the same (*α*_*K*_ = *α*_*B*_ = 0.99, *ρ* = 0.8). The simulations show that the change in the covariance matrix and subsequently biasing the distribution only changes how the learning trajectories evolve, not the decay trajectories; for all simulations the decay trajectories follow a straight line back to the origin due to the symmetry of the decay rates.
(EPS)

S6 Fig The influence of the retention rates on simulated learning decay trajectories. The simulation of the asymmetric model for the learning (colored traces) and decay trajectories (black traces) in primitive gain space for different retention rates for position- and velocity-based learning: symmetric rates **(A)** (*α*_*K*_ = *α*_*B*_ = 0.96, *σ*_*K*_ = *σ*_*B*_ = 0.5, *η* = 1.5 x 10^−4^, *ρ* = 0.8), asymmetric, with the velocity-based retention higher than the position-based **(B)** (*α*_*K*_ = 0.96, *α*_*B*_ = 0.97, *σ*_*K*_ = *σ*_*B*_ = 0.5, *η* = 1.53 x 10^−4^, *ρ* = 0.8) and asymmetric, with the position-based retention higher than the velocity-based **(C)** (*α*_*K*_ = 0.97, *α*_*B*_ = 0.96, *σ*_*K*_ = *σ*_*B*_ = 0.5, *η* = 1.53 x 10^−4^, *ρ* = 0.8). These simulations show that any asymmetry in the ratio of the retention rates mainly affects the shape of the decay trajectory (black traces, curving towards the axis with greater retention), with little difference in the learning trajectories (colored traces).
(EPS)

S7 Fig Simulation of the asymmetric viscoelastic primitive model for adaptation to a velocity biased force-field perturbation. **(A)** Adaptation (orange trace) and decay (black trace) to a velocity biased force-field (vcFF, a greater velocity and smaller position dependence). This learning goal is represented by the point [0.53, 0.85] in gain space. The parameter values are the same as those used in the simulations presented in Fig 4 (*α*_*K*_ = 0.9532, *α*_*B*_ = 0.9602, *σ*_*K*_ = 0.3758, *σ*_*B*_ = 0.3280, *η* = 1.5 x 10^−4^, *ρ* = 0.50). **(B)** Normalized decay of position- and velocity-dependent gains for vcFF training. Similar to the simulations for ucFF and pcFF adaptation, the position-based learning for the vcFF perturbation decays faster than velocity.
(EPS)

S8 Fig Exponential fits to position and velocity-based learning for ucFF and pcFF adaptation decay. **(A)** A standard exponential model with rate and offset parameters fit to the decay of the velocity- and position-based learning for ucFF training (data presented in Fig 5C). A time constant of 9.4 ± 1.8 was estimated for position-based adaptation and 12.1 ± 2.0 trials for velocity-based learning. **(B)** The exponential model was fit to the decay of the velocity- and position-based learning for pcFF training (data presented in Fig 5D). A time constant of 5.7 ± 0.6 was determined for position-based learning and 9.3 ± 0.9 trials for velocity-based adaptation. As in S1 Fig and S2 Fig, values represent the mean ± the standard deviation from bootstrapping.
(EPS)
